# Surveillance for *Chlamydia trachomatis* variants escaping detection with the Aptima Combo 2 assay in Canada from 2019 to 2021

**DOI:** 10.1128/spectrum.02062-24

**Published:** 2025-01-23

**Authors:** Felicia Roy, Jennifer Beirnes, Jason J. LeBlanc, Suzanne Gibbons, Aida Sivro, Alberto Severini, On behalf of the Canadian Public Health Laboratory Network (CPHLN) Chlamydia Variant Working Group

**Affiliations:** 1 National Microbiology Laboratory Branch, Public Health Agency of Canada, Winnipeg, Manitoba, Canada; 2 Division of Microbiology, Department of Pathology and Laboratory Medicine, Nova Scotia Health, Halifax, Nova Scotia, Canada; 3 Departments of Pathology, Medicine (Infectious Diseases), and Microbiology and Immunology, Dalhousie University, Halifax, Nova Scotia, Canada; 4 Department of Medical Microbiology and Infectious Diseases, Faculty of Health Sciences, University of Manitoba, Winnipeg, Manitoba, Canada; 5 Centre for the AIDS Programme of Research in South Africa (CAPRISA), Durban, South Africa; 6 Department of Medical Microbiology, University of KwaZulu-Natal, Durban, South Africa; Nevada State Public Health Laboratory, Sacramento, California, USA; Massey University, Wellington, New Zealand; Royal Women's Hospital, Parkville, Victoria, Australia

**Keywords:** *Chlamydia*, variant, mutation, surveillance, sequencing, diagnostic, Aptima, Panther

## Abstract

Nucleic acid amplification tests (NAATs) are the method of choice for *Chlamydia trachomatis* diagnosis, but these strategies are susceptible to target site mutations. *C. trachomatis* variants escaping detection with the Aptima Combo 2 (AC2) assay on the Hologic Panther instrument from 23S rRNA mutations have been reported in Nordic countries, England, Japan, and the United States. Given the potential for false negative results, this study investigated whether strains of *C. trachomatis* with AC2 target site mutations were present in Canada. Surveillance was conducted in Canadian laboratories from 2019 to 2021. Specimens suspected of AC2 target site mutations included those with low-value detections on the AC2 assay, with subsequent high-value detections on the Aptima Chlamydia Trachomatis (ACT) assay used for confirmatory testing. Specimens with AC2/ACT discrepant results were subjected to sequencing of the AC2 target (i.e., 23S rRNA). Sequencing revealed 15 (4.8%) diagnostic escape variants which were carrying either C1514T, G1523A, or G1526A mutations. All specimens with *a* diagnostic escape mutation were detected with a reformulated version of the AC2 assay. Overall, while the prevalence of *C. trachomatis* variants was rare, their presence in the Canadian population supports the use of the new AC2 kit formulation and the need for ongoing genetic surveillance for NAAT-based assays.

**IMPORTANCE:**

Molecular tests are commonly used for the detection of sexually transmitted infections (STIs) like *Chlamydia trachomatis*. Mutations impacting *C. trachomatis* molecular target detection on the Hologic Panther AC2 assay have been reported in several countries, raising concerns about potential false negative results. This study showed *C. trachomatis* target detection failures in specimens submitted for *C. trachomatis* testing in Canadian laboratories from 2019 to 2021. A reformulated version of the AC2 molecular test is now available that can identify *C. trachomatis* strains harboring target site mutations that were impacted by the previous test formulation. While target site mutations were rare in Canada, revealing their presence is important to ensure accurate molecular detection of *C. trachomatis* with existing testing methods. This study supports ongoing genetic monitoring of *C. trachomatis* molecular test target sites, as well as the use of the reformulated test to avoid false negative results and subsequent transmissions.

## OBSERVATION

*Chlamydia trachomatis* (CT) remains the most prevalent bacterial STI, with an estimated 130 million new infections reported globally each year (1). Clinical sequelae can include pelvic inflammatory disease, ectopic pregnancy, and infertility; however, as asymptomatic infections are common, many CT infections go unnoticed without routine screening (2). Nucleic acid amplification tests (NAATs) like real-time PCR and transcription-mediated amplification (TMA) have been the most frequent NAATs used in clinical and public health laboratories, often with concomitant detection of *Neisseria gonorrhoeae* (or gonococcus [GC]) (3, 4). Like any molecular method (5), NAATs for CT/GC are designed to target conserved genomic regions, but mutations can arise over time with microbial evolution. If sequence mismatches occur within the NAAT target site, reduced sensitivity or detection failure could ensue (5–10).

False negative CT results occurred on a commonly used commercial NAAT for CT/GC testing, the Hologic Aptima Combo 2 (AC2) assay on the Panther instrument (Hologic Inc., San Diego, CA, USA) (11–17). The culprit termed the Finnish new variant of CT (FI-nvCT) harbored a 23S rRNA C1515T mutation, corresponding to the target site of the acridinium probe used for the CT detection. The emergence of FI-nvCT causing false negative results prompted surveillance in many countries (11–17). This variant along with other diagnostic escape mutations (i.e., C1514T, A1518G, C1522T, G1523A, and G1526A) were identified in Finland, and other Nordic countries (i.e., Sweden, Norway, and Denmark), England, Japan, and recently the United States (11–17). This study reports surveillance for CT diagnostic escape mutants on the AC2 assays in Canada from June 2019 to August 2021, prior to the introduction of a new reformulated assay with a novel CT probe.

Swab or urine specimens in Aptima Specimen Transport Media were submitted to the National Microbiology Laboratory (NML, Winnipeg, MB) from participating Canadian provincial public health laboratories across five Canadian provinces (i.e., Alberta [AB], British Columbia [BC], Ontario [ON], Quebec [QC], and Saskatchewan [SK]). As per previous recommendations (12), eligible samples were those with equivocal results on the AC2 CT/GC assay on the Panther instrument with relative light unit (RLU) values between 25 and 99, that were positive (>100 RLU) following reflex testing on the Aptima Chlamydia Trachomatis (ACT) assay (which detects an alternative CT-specific target, the 16S rRNA). All eligible samples were queued for 23S rRNA gene sequencing targeted by the AC2 assay. For each specimen, 500 µL were processed as per manufacturer instructions using the DNA and Viral NA Large Volume Kit (Roche Diagnostics, cat# 06374891001) on a MagNAPure 96 instrument (Roche Diagnostics, Laval, QC), and nucleic acids were eluted in 50 µL. For 23S rRNA gene sequencing, a nested RT-PCR protocol was provided by Dr. Damon Getman (Scientific Director), Hologic Inc. The first round RT-PCR was performed with 5 µL of template in 25 µL reactions using primers and concentrations defined in Table S1, and the buffer and enzymes from the Invitrogen SuperScript III One-Step RT-PCR System with Platinum Taq (Invitrogen, Cat. # 12574026). Amplification conditions were as follows: reverse transcription (55°C, 30 min), initial denaturation (94°C, 2 min), followed by 40 cycles of denaturation (94°C, 15 s), annealing (56.5°C, 30 s), and extension (68°C, 1 min). After final elongation (68°C, 5 min), amplicons were held at 4°C until use. The second round of PCR was performed in 25 µL reactions consisting of primers (Table S1), 1 µL of template from the first-round reaction, and buffers and enzymes from the OneStep Ahead RT-PCR kit (Qiagen Inc., Cat. # 220211). Cycling conditions were as follows: initial denaturation (95°C, 5 min), followed by 40 cycles of denaturation (95°C, 10 s), annealing (56.5°C, 10 s), and extension (72°C, 10 s). Final elongation (72°C, 2 min) was followed by holding at 4°C until use. The resulting amplicons were visualized using the QIAxcel Advanced system (Qiagen, Cat. # 9001941), according to manufacturer instructions. Sanger sequencing was performed by the NML DNA Genomics Core Facility. Specimens yielding sequencing results were analyzed using the Vector NTI 11.5.3 and MEGA6 (18), with alignments to reference CT serovars D, E, and J using the National Center for Biotechnology Information (NCBI) Genbank database accession numbers CP007131.1, HE601870.1, and CP017741.1, respectively. Additionally, nucleic acids were subjected to an in-house real-time quantitative PCR (qPCR) targeting the Chlamydia trachomatis cryptic plasmid as previously described (19) and crossing point (Cp) values were recorded.

Of 313 samples submitted for sequencing, sequencing failures occurred in 146 (46.6%) (Table 1). This high proportion of specimens with low sequencing efficiency has been reported by others for AC2 equivocal specimens and is likely due to specimens with low bacterial loads falling below or near the limit of detection of the sequencing reactions (15, 20). Supporting this hypothesis, 93 (63.7%) of the 146 specimens with sequencing failures were negative for the cryptic plasmid (CP) target, with Cp values ≥40 using an in-house real-time qPCR. The Cp values of the diagnostic escape variants were significantly lower (*P* < 0.0001) than those of strains with wild-type sequences, suggesting higher bacterial loads were present (Fig. S2). In total, 190 samples were CP positive, 115 were negative, and 8 were NSQ as tested by in-house real-time quantitative PCR.

**TABLE 1:** Summary of 23S rRNA sequencing and variants detected

Description	Province					
	AB	BC	ON	QC	SK	Total
C1515T (FI-nvCT)	0	0	0	0	0	0
C1514T	5	0	3	0	0	8
C1522T	1	0	0	0	0	1
G1523A	1	0	0	0	0	1
G1526A	5	0	0	0	0	5
WT (serovar D)^*a*^	0	0	0	5	0	5
WT (serovar E)^*a*^	6	10	8	18	5	47
WT (serovar J)^*a*^	39	8	28	20	5	100
Sequencing failed	49	6	35	33	23	146
Total	106	24	74	76	33	313

^*a*^ Wild-type (WT) sequences for CT most closely resembling serovars D, E, and J in the 23S rRNA target region were derived from Genbank accession numbers CP007131.1, HE601870.1, and CP017741.1, respectively.

Of the 167 successfully sequenced specimens, 152 (91.0%) contained wild-type 23S rRNA sequences. These sequences most closely resembled serovars D (5 [3.3%]), E (47 [31.0%]), and J (100 [65.8%]) within the target region (Fig. S1; Table 1). The remaining 15 sequences harbored mutations known to cause diagnostic escape with the AC2 assay (11–17): C1514T (*n* = 8), G1526A (*n* = 5), and one each with G1523A or C1522T (Fig. S1; Table 1). G1523A, C1514T, and C1522T were previously reported by others (13–15, 21), but the G1526A mutation was more recently described in the United States (11). No sequences corresponded to the other mutations causing detection failures for the AC2 assay, e.g., C1515T (16, 17) or A1518G (11).

While the proportion of CT diagnostic escape variants during the study period is estimated to be low (<0.01%), this may be underestimated due to the inherent biases of specimen selection (i.e., focus on equivocal samples) (11, 12), the voluntary submissions from participating laboratories, and the sensitivity limitations of sequencing methods. Parallel testing of all specimens with the AC2 and ACT assays (or between other NAATs) could be a solution to identify additional CT variant cases; however, this strategy was not possible with the high volumes of CT/GC testing occurring in clinical and public health laboratories. Regardless, the low proportion of CT variants seen in this study was consistent with other countries using a similar surveillance strategy (11–17). For example, the prevalence of AC2 diagnostic escape mutants (i.e., C1514T and G1523A) was <0.003% in England (13). Despite the low prevalence of diagnostic escape mutants observed in this and other studies, there was an ongoing risk of further transmissions. As seen with the Swedish novel CT variant (SW-nvCT) with NAATs used at the time, unrecognized circulation of variants can lead to significant disease spread (5–7). Hologic Inc. recently redesigned the AC2 assay to include an additional CT detection probe falling outside the region affected by the recent 23S rRNA diagnostic escape variants (11, 20, 21), which is a common strategy to mitigate NAAT target site mutations (5, 22–25). Following retesting with the reformulated AC2 assay, all specimens with CT variants from this study were found to be positive (Table 2).

**TABLE 2:** Analytical characteristics of the 15 diagnostic escape variants detected in this study

NML identifier	Results				
	23S rRNA mutation	AC2 (RLU)^*a*^	ACT (RLU)^*a*^	qPCR (Cp)^*a*^	Reformulated AC2 (RLU)^*a*^
CLM20-1137	C1514T	70	7258	27.28	556
CLM20-1139	C1514T	82	7081	20.04	393
CLM21-1417	C1514T	38	6641	23.62	457
CLM21-1442	C1514T	33	6936	27.63	417
CLM19-5277	C1514T	53	6109	29.29	360
CLM19-5283	C1514T	26	6078	30.55	401
CLM19-5517	C1514T	90	7600	20.87	396
CLM21-1455	C1514T	47	9112	27.64	405
CLM22-0087	C1522T	26	7013	28.39	423
CLM19-5278	G1523A	88	8110	33.73	594
CLM21-1393	G1526A	32	6733	29.46	533
CLM21-1402	G1526A	53	6327	25.60	591
CLM21-1405	G1526A	53	6903	24.35	549
CLM22-0090	G1526A	54	6749	30.91	650
CLM22-0089	G1526A	38	6799	30.35	649

^*a*^ The AC2, ACT, and reformulated AC2 assay were performed on the Panther instrument, where the qPCR represents an in-house real-time PCR targeting the cryptic plasmid. While eligibility criteria were followed for testing, these data were not submitted by the participating laboratory.

Overall, the presence of mutations in the AC2 target region emphasizes the importance of conducting regular genomic surveillance to monitor the evolution and diversity of Chlamydia Trachomatis serovars circulating within the country.

## Supplementary Material

Reviewer comments

## SUPPLEMENTAL MATERIAL

Supplemental table and figures spectrum.02062-24-s0001.docx Table S1; Fig. S1 and S2.

OPEN PEER REVIEW reviewer-comments.pdf An accounting of the reviewer comments and feedback.

ASM does not own the copyrights to Supplemental Material that may be linked to, or accessed through, an article. The authors have granted ASM a non-exclusive, world-wide license to publish the Supplemental Material files. Please contact the corresponding author directly for reuse.
