# Supplementary material for: Surveillance for Chlamydia trachomatis variants escaping detection with the Aptima Combo 2 assay in Canada from 2019 to 2021
Source: Microbiol Spectr. 2025 Jan 23;13(3):e02062-24. doi: 10.1128/spectrum.02062-24 (PMC11878061; doi:10.1128/spectrum.02062-24)
Supplement: Supplemental table and figures — Table S1; Fig. S1 and S2. [file spectrum.02062-24-s0001.docx]

**Supplemental materials**:

**Table S1**. Oligonucleotides used for PCR amplification and sequencing of the *C. trachomatis* 23S rRNA.

| Name | Sequence  (5’ to 3’) | Final concentration  [µM] | Amplicon Length  (bp) |
| --- | --- | --- | --- |
| CtrB(+)1078 | GTGGGTAAGGAAGTGATGATTCG | 0.4 | 695  Outer Primer Pair |
| CtrB(-)1773 | GCTCCTATCGTTCCATAGTC | 0.4 |  |
| CtrB(+)1243 | GAGAGCGTAGTATTCAGCAGAG | 1.0 | 516  Inner Primer Pair |
| CtrB(-)1759 | CCATAGTCACCCTAAAAGGCTC | 1.0 |  |
| CtrB(+)1403 | CCTAAGTTGAGGCGTAAC | 1.0 | Sequencing primer |
| CtrB(-)1640 | GTACGGTCACCATCAACAGC | 1.0 | Sequencing primer |

**Figure S1.** Alignment of 23S rRNA sequence variants detected in this study. Mutations in the 23S rRNA associated with diagnostic escape with the Aptima AC2 assay variants are shown in red, and numbering is based on the *E. coli* 23S rRNA. In blue are polymorphisms not associated with diagnostic escape but found in wild-type (WT) sequences for CT serovars D, E, and J (derived from Genbank accession numbers CP007131.1, HE601870.1, and CP017741.1, respectively).


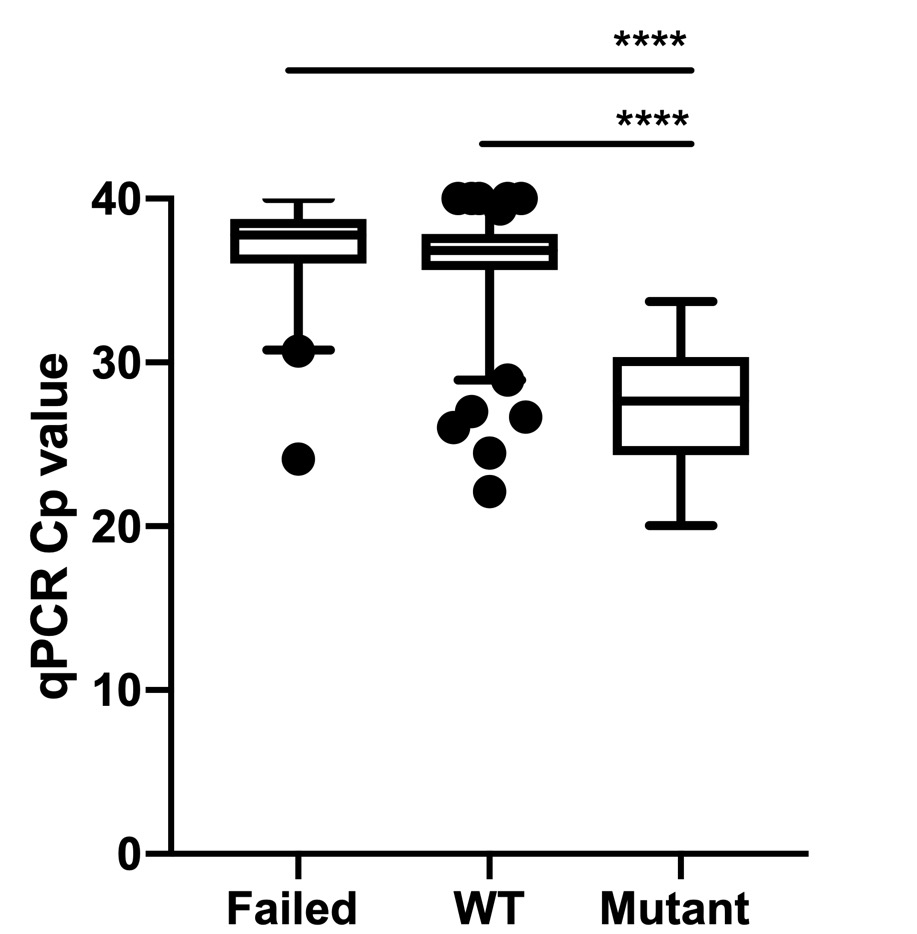


**Figure S2.** ‘Box and Wiskers’ graph of the cryptic plasmid qPCR crossing point (Cp) values for specimens that could not be sequenced at the 23S rRNA target (Failed), that produced a wild type 23S mRNA sequence (WT) or that produced an escape mutant sequence (Mutant). The mutant group was significantly different from both the failed and WT groups (p<0.0001), as determined by Kruskal-Wallis test followed by Dunn’s multiple comparison test. Plotting and statistical analysis was performed using Prism 8 (GraphPad Software LLC, San Diego, California, USA). Boxes represent median and interquartile range; whiskers represent 5–95 percentiles. ****p<0.0001.
